# Negative online news articles are shared more to social media

**DOI:** 10.1038/s41598-024-71263-z

**Published:** 2024-09-16

**Authors:** Joe Watson, Sander van der Linden, Michael Watson, David Stillwell

**Affiliations:** 1 Psychometrics Centre, Judge Business School, University of Cambridge, Cambridge, UK; 2 Department of Psychology, University of Cambridge, Cambridge, UK; 3 Department of Informatics, King’s College London, London, UK; 4 Organisational Behaviour Group, Judge Business School, University of Cambridge, Cambridge, UK

**Keywords:** Negativity bias, Online news, Social media, Human behaviour, Psychology and behaviour

## Abstract

Prior research demonstrates that news-related social media posts using negative language are re-posted more, rewarding users who produce negative content. We investigate whether negative material from external news sites is also introduced to social media through more user posts, offering comparable incentives for journalists to adopt a negative tone. Data from four US and UK news sites (95,282 articles) and two social media platforms (579,182,075 posts on Facebook and Twitter, now X) show social media users are 1.91 times more likely to share links to negative news articles. The impact of negativity varies by news site and social media platform and, for political articles, is moderated by topic focus, with users showing a greater inclination to share negative articles referring to opposing political groups. Additionally, negativity amplifies news dissemination on social media to a greater extent when accounting for the re-sharing of user posts containing article links. These findings suggest a higher prevalence of negatively toned articles on Facebook and Twitter compared to online news sites. Further, should journalists respond to the incentives created by the heightened sharing of negative articles to social media platforms, this could even increase negative news exposure for those who do not use social media.

## Introduction

Most adults now consume news digitally. In the UK, 68% of adults report that they access news through one or more online sources^1^. In the US, 86% of adults report sometimes or often accessing news using a digital device^2^. Digital devices allow news consumption through social media platforms, which 49% of US adults use to obtain news at least sometimes, and news websites or apps, used by 67% of US adults^2^. Extensive evidence demonstrates that negativity influences news engagement on dedicated news sites^3^ and social media platforms^4^. However, there is a need for more comprehensive research regarding whether negative content from external news sites is more likely to be introduced to social media through user posts containing article links. Many consumers obtain news via these posts^5^. If the sharing of online news articles to social media was influenced by negativity, it would lead to a higher prevalence of negatively toned news articles on social media and incentivise journalists to produce more negative content.

Previous research largely highlights the deleterious effects of negative news exposure. These effects include emotional responses like sadness^6^, anger^7^, stress^8^ and anxiety^9^. Studies also suggest that reading neutral or positive news is devoid of psychological or physiological costs^10^, and interventions focused on delivering positive news content promote mental health^11^. However, it should be recognised that negative content can encourage information-seeking behaviours^7,12^ and, in the realm of political communication, potentially foster a deeper understanding than positive messages^13–15^.

Despite the adverse consequences of exposure to negative content, humans often appear predisposed towards it. This can be understood through the concept of negativity bias^16^, which refers to the innate tendency to assign greater significance to negative information than positive information^7,17^. The literature strongly suggests that negativity bias influences the consumption of conventional news media. News consumers have long been found to favour reading negative news articles^18^, which prove more eye-catching and digestible^19^. This behaviour occurs even among online news readers who report a desire for more positivity in the news media^3^. Additionally, negative words in news headlines significantly increase click-through rates to the full news articles^20^.

While social media posts^21,22^, including those about certain news events^23^, lean positive, studies suggest that the negativity of news-focused social media posts increases their subsequent dissemination. Analysis of posts from the official Twitter (now, X) accounts of news organisations has shown that negative phrasing leads to heightened retweeting behaviour^4^. Schöne, Parkinson and Goldenberg^24^ also found that negativity predicts spread among tweets posted in response to positive and negative political situations. Moreover, users are more likely to comment on Facebook posts from politicians containing words indicating negative emotions^25^. Nevertheless, there are exceptions. For instance, Jung and colleagues^26^ analysed over 4,400 Facebook posts from various US and UK news sources, concluding that post sentiment had no significant effect on re-posting. Additionally, Camaj, Çela and Rexha^27^ observed that negative Facebook posts from the Albanian and Kosovan news media during electoral campaigns received fewer re-posts, reactions, and user comments.

There has been some prior investigation into the effect of a news article’s negativity (and positivity) on the likelihood of it being introduced to social media, yielding mixed results. Henn and Posegga^28^ found that the negativity of a news article’s title increases its spread on Reddit. Similarly, De Leon and Trilling^29^ identified that negativity amplifies the sharing of political content from Mexican news sites to social media. We also note that Heidenreich and colleagues^30^ explored the effect of article negativity on reactions to posts made by news organisations’ official Facebook accounts that concerned EU political news articles. The negativity of an article was found to increase the number of Facebook re-posts and reactions, but not comments^30^. However, Trilling, Tolochko and Burscher^5^ found positivity to be a stronger predictor of Facebook and Twitter shares than negativity for a sample of Dutch news articles. Bakshy and colleagues^31^ also found that linked content evoking positive emotions had a larger cascade depth on Twitter. However, they did not extend their analysis to consider negative emotions. Relatedly, Berger and Milkman^32^ established that the positivity of an online New York Times article increased the likelihood of it being featured on the site's 'most emailed' list. Beyond providing mixed findings, each of these studies is limited in scope, for instance, by focusing solely on an individual social media platform, newspaper, country, or news topic. Thus, we aim to extend prior research by conducting an analysis of large-scale data comprising online news articles, Facebook posts, and tweets (see, “Methods”) to investigate the primary hypothesis:

H1: Negative online news articles are shared more to social media

H1 addresses a general propensity to share negative information, which overlooks individual differences. While negativity bias has been observed among those of different ages (from young children^33,34^ to adults^35^) and in a broad array of settings^36,37^, the extent of this bias may vary considerably^38^. Those farther to the right of the political spectrum possess heightened *self-reported* reactions to negative stimuli^39^, although reports of an association between *physiological* response and political ideology^40,41^ have been disputed^39,42,43^.

Multiple alternate factors may also influence individuals’ engagement with online information, including its alignment with their pre-existing beliefs^44,45^ and political identity^46,47^. This could be attributable to individuals associating more with members of their political in-group^46^, favouring pro-attitudinal online content^44,45,48^. Such in-group favouritism might manifest in individuals disseminating fact-checking messages that support their political in-group^49^ and social media posts by news organisations containing in-group language^46^. While in-group alignment is evident, opposition to an out-group may have an even more pronounced effect.

Existing research has identified out-group animosity as a robust predictor of online behaviours, including disseminating fake political news articles to discredit out-group politicians^39^. Notably, Rathje, Van Bavel and Van der Linden^46^ found that out-group language was a key predictor of the number of re-posts received by social media posts, often surpassing the influence of in-group language and even negativity. Their research also indicated that posts concerning out-groups might generate engagement by eliciting emotions such as anger and outrage^46^. However, the formal interaction between out-group referencing and the negativity of news content still requires investigation. We seek to address this gap by examining the secondary hypothesis:

H2: Article negativity is a stronger determinant of social media sharing for content about political out-group (versus in-group) members

## Results

We analysed news articles from the Daily Mail, Guardian, New York Times, and New York Post published between 2019 and the end of 2021, along with all social media posts referencing these articles on Facebook or Twitter (see, “Data”). This resulted in 8 distinct datasets: one for each news site and social media platform combination.

### Article negativity and sharing to social media

**Fig. 1:**
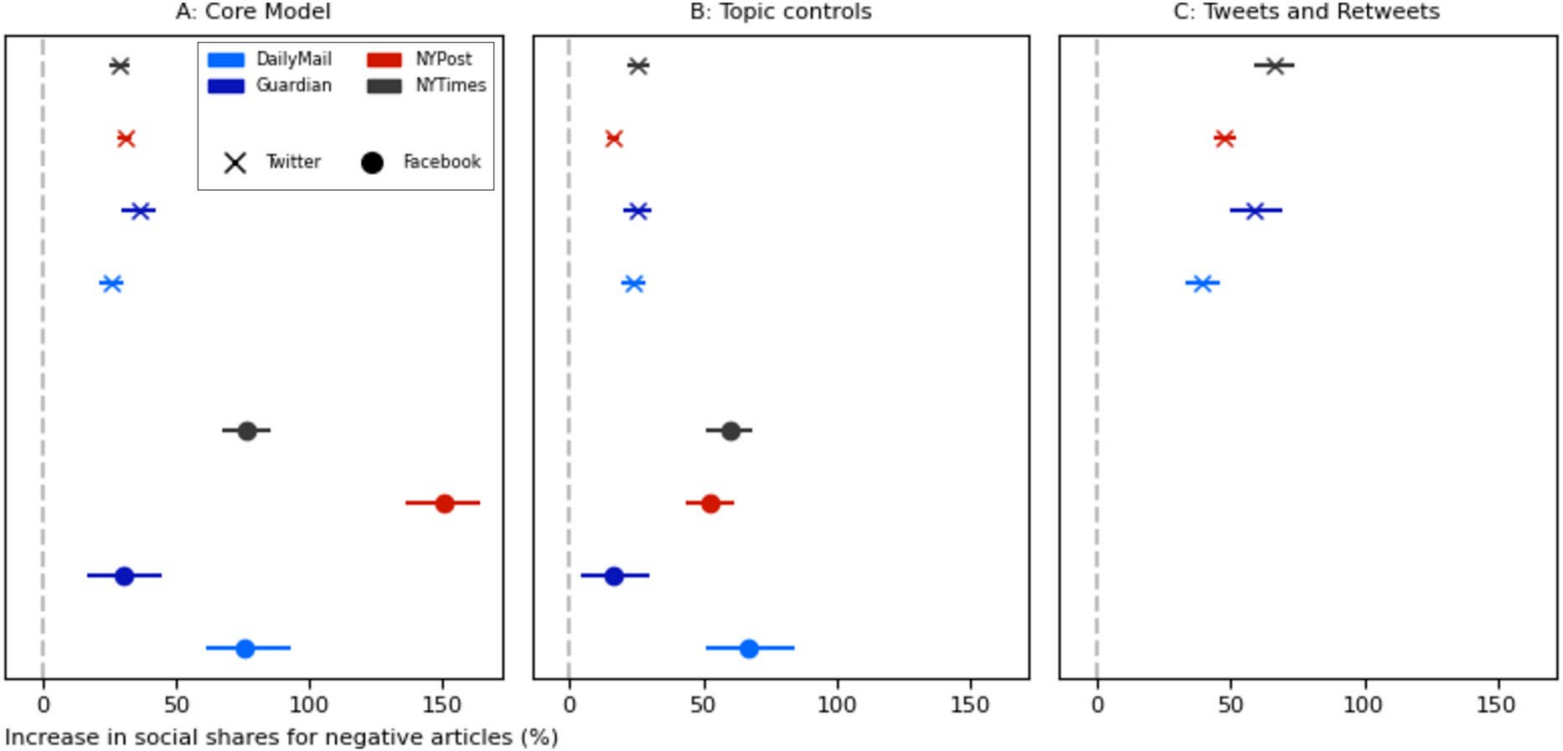
Point estimates and error bars (95% confidence intervals) for the effect of article negativity on log(+ 1) article shares, converted into percentage increases to enhance readability (SI9). Treatment effect values and n values are provided in SI1. The effect of article negativity on shares is positive and significant (p < 0.05) across every dataset when applying our core model (**A**), the same model with added news article topic controls (**B**), and a model predicting tweets plus retweets (**C**).

Doubly robust estimation (DRE) was employed to predict the (log + 1) count of social media posts linking to a news article, based on the binary negativity label assigned to the article through a dictionary-based process, and numerous controls for article characteristics (see, “Methods”). Applying this approach to 1000 bootstrapped samples from each Facebook dataset gave average treatment effect means of 0.265 to 0.916 (SI 1, Fig. 1A), implying that negative news articles are shared 30% to 150% more to social media (when applying the conversion approach presented in SI9). Using the same method on Twitter samples produced treatment effect means between 0.232 and 0.309 (SI1, Fig. 1A, 26% and 36% more shares). Lastly, an aggregate finding was obtained by predicting both Facebook and Twitter shares for articles from all news sites. This produced a treatment effect mean of 0.646 (SI2, 91% more shares). Thus, findings from our core model indicate that the negativity of an article is an important factor in its sharing to social media.

#### Robustness

The stability of results produced through our core model was confirmed by comparing them to those from multiple alternate approaches (Fig. 1B, Fig. SI.3.1A–C). These approaches were a multiple regression (MR) model, a propensity score (PS) model, a DRE model using an alternate treatment variation, and the core DRE model with additional topic controls (see, “News Article Data”). Application of all robustness check models across every dataset shows negative articles to receive significantly more shares on social media, with results from these models only differing from the core model in 4 (of 32 possible) instances (SI1).

#### Variation

Core model findings revealed significant differences in the effect of negativity on the sharing of articles from left- and right-leaning news sites to Facebook, although no differences were observed for Twitter. Specifically, the effect of article negativity on Facebook sharing was higher for right-leaning papers than left-leaning papers (categorised according to AllSides^50^). Right-leaning papers had aggregated mean treatment effects of 0.799 (122% more shares), while left-leaning papers had 0.531 (70% more shares, SI2). This difference aligns with previous research indicating that individuals on the political right have stronger reported (as opposed to physiological) responses to negative stimuli^39^. Emotive tweets from the official accounts of conservative news organisations have also been found to elicit greater engagement, unlike tweets from their liberal counterparts^51^. Moreover, the larger negativity coefficient for right-leaning papers may be associated with the heightened algorithmic amplification observed for such news sources^52^.

Analyses also suggested a difference in the influence of negativity on news article sharing between social media sites, with a significantly larger impact on Facebook than Twitter sharing for articles from the Daily Mail, New York Post and New York Times. Aggregated models predicting the sharing of articles from all papers on Facebook or Twitter showed that Facebook users were more likely to share negative articles (treatment effect 0.682; 98% more posts) than Twitter users (treatment effect 0.296; 34% more tweets, SI2). Several factors may underlie this disparity, including differences in demographics and usage patterns between Facebook and Twitter users^53,54^ and the potential presence of errors in social media sharing data (which were captured using distinct methods, “Social Media Data”, “Discussion”). However, efforts were made to mitigate data collection inaccuracies (see, “Discussion”), and there is an overlap between the user bases of Facebook and Twitter^55^. Thus, we speculate whether the algorithms underlying the Facebook platform act to promote the sharing of negative content to a greater extent than those on Twitter.

The reach of negative news articles could be extended by events occurring after their initial introduction to social media through user posts, including whether these posts are themselves re-posted. We explore this possibility using Twitter data, adapting our original model to use a broader measure of news article sharing that encompasses both the original tweets and their retweets (Fig. 1C). Application of this expanded model to each news site sample consistently yielded treatment effect means that were significantly higher than when predicting the number of original tweets alone (SI1). An aggregated model applied to data from all news sites produced an effect of 0.478 (61% more tweets plus retweets), significantly more than the effect of article negativity on sharing through tweets alone (0.295, 34% more tweets only, SI2). Moreover, tweets sharing negative articles are significantly more likely to be retweeted (effect = 0.022, 2% more retweets, SI4). Hence, Guess and colleagues’^56^ discovery that the inclusion of re-posts in Facebook user feeds increases the spread of political news articles may stem, in part, from the impact of re-posts on negative (political) news article dissemination.

**Fig. 2:**
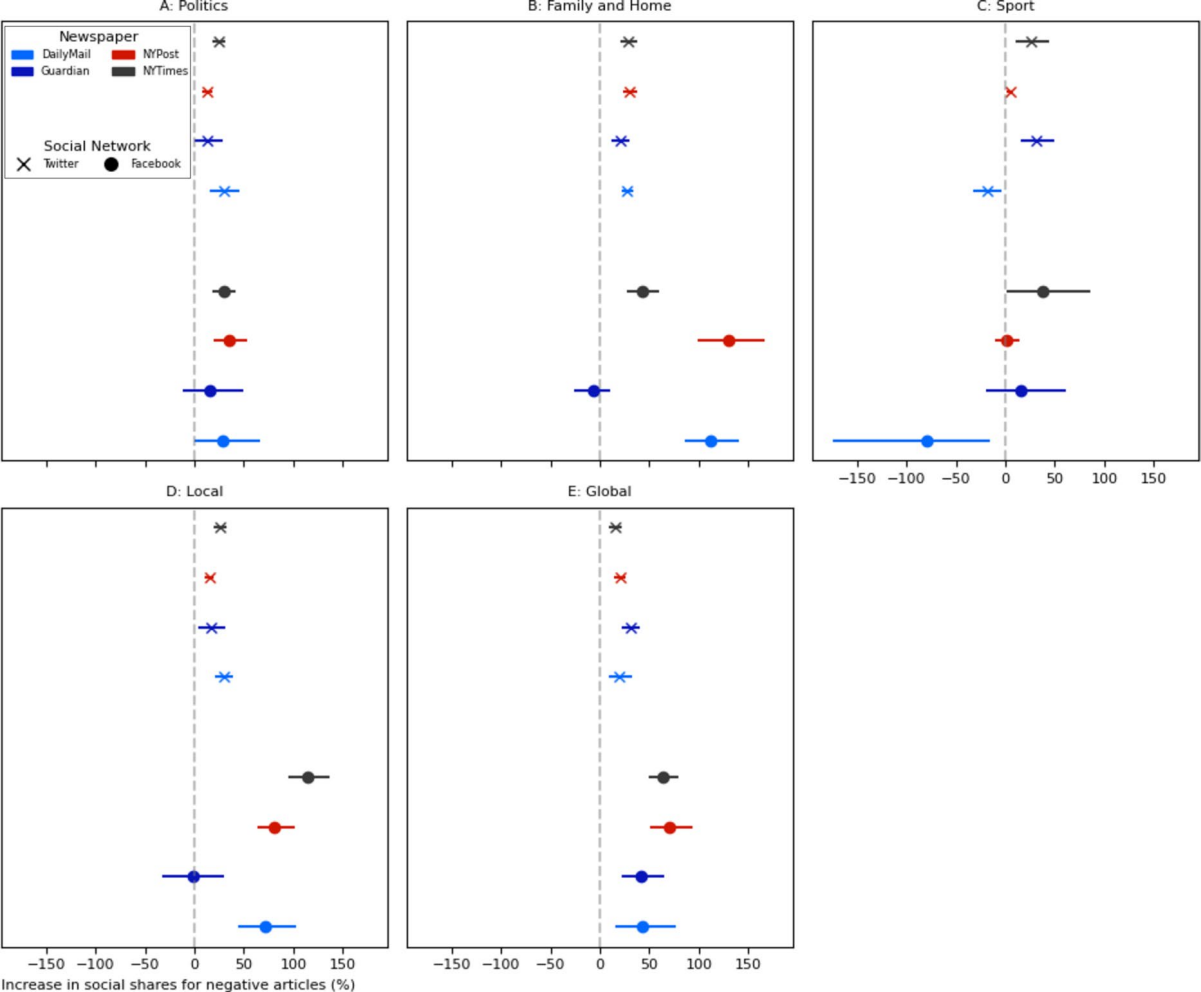
Point estimates and error bars (95% confidence intervals) for the effect of article negativity on log(+ 1) article shares across various topics, converted into percentage increases (SI9). Treatment effect values and n values are provided in SI5. The impact of negativity shows variation across subsamples comprising articles categorised as concerning different topics.

We also explored whether the influence of negativity bias is affected by news article topic (established through topic modelling, “News Article Data”), as indicated by prior research^20,48^. MR models controlling for key article characteristics applied to topic-based data subsets showed that negative articles on global news, local news, family and home, and political topics were shared more (in at least 7 of the 8 newspaper and social media site combinations for each topic, Fig. 2, SI5). Conversely, the relationship between sports article negativity and sharing to social media sites was mixed, with significant coefficients indicating both positive and negative effects.

### Article negativity, out-group content, and sharing to social media

**Fig. 3:**
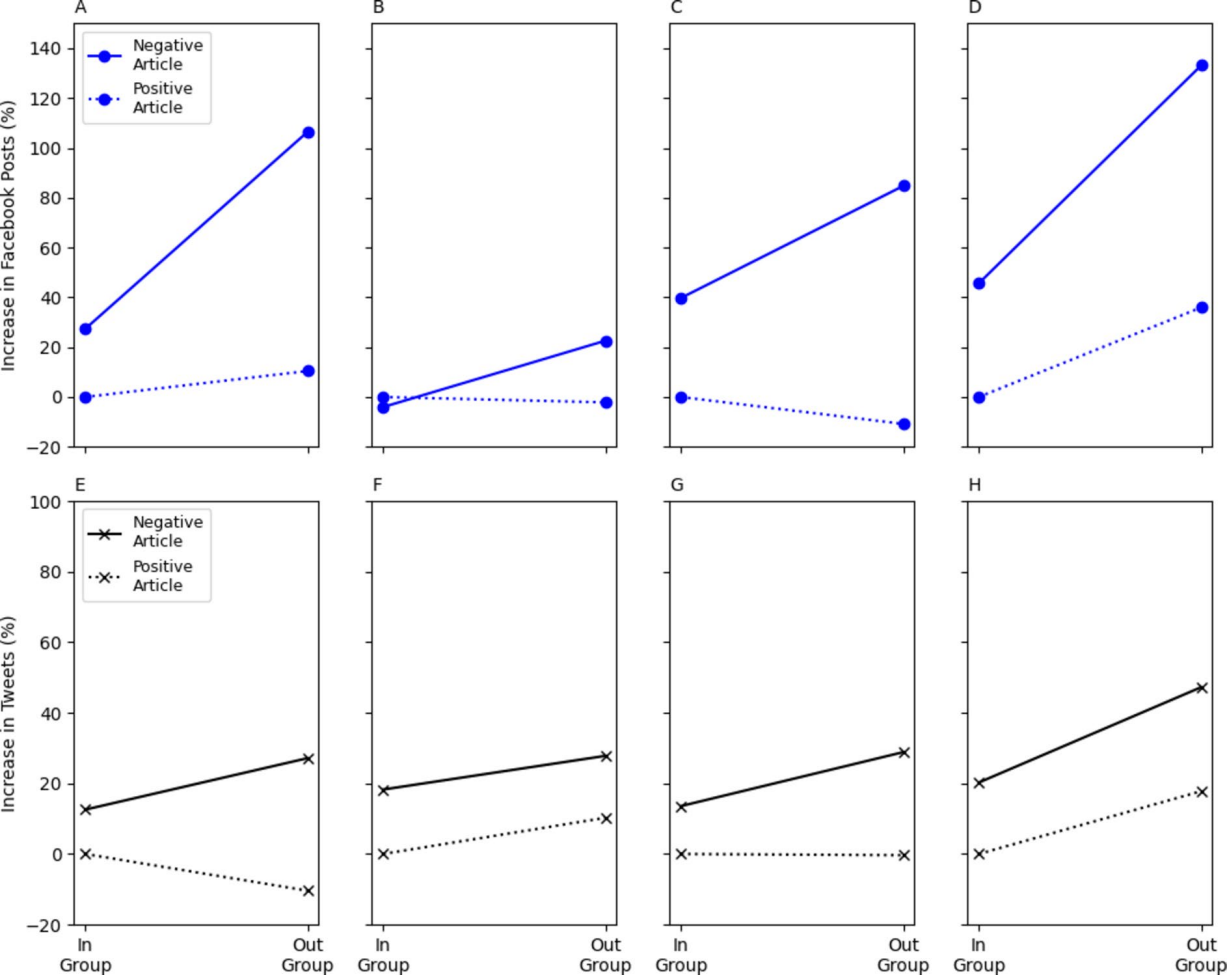
The influence of out-group membership, article negativity, and their interaction. Point estimates obtained using MR have been transformed into percentage increases for legibility (SI9). Subplots pertain to posts or tweets about articles from the Daily Mail (**A,E**), Guardian (**B,F**), New York Post (**C,G**) and New York Times (**D,H**). There is a positive interaction effect between article negativity and referencing an out-group in 7 out of 8 datasets (**A–E,G,H**). This positive interaction effect is statistically significant (p < 0.05) in 5 instances (**A-C,E,G**). Results are presented in tabular format in SI6.

We employed MR models with an interaction term between binary variables showing whether the article was negative (instead of positive) and predominantly concerned political out-group (as opposed to in-group) concepts (see, “News Article Data”). Consistent with Rathje, Van Bavel and Van der Linden^46^, out-groups are determined in relation to the political stance of the news site. These models were applied to our eight samples, subset to retain only articles that predominantly referenced a political out- or in-group (News article data, SI6, Fig. 3). Additionally, we constructed an aggregated model predicting Facebook and Twitter shares of articles from all news sites. This showed that both negativity and out-group referencing have a positive and significant impact on sharing to social media, and that there is a significant interaction between the two (SI2). This interaction effect suggests that articles that predominantly reference out-group politicians and are negative yield a greater increase in shares than would be obtained from either factor individually.

## Discussion

The findings we present highlight the influence of negativity on the introduction of online news articles to social media. This observation holds across multiple alternative models (see, “Robustness”), although the influence of negativity varies based on factors including whether Facebook or Twitter shares are predicted (with higher treatment effects identified for the former, see, “Variation”). Our exploration of H2 also indicates that the tendency of social media users to share negative news is moderated by the article's political focus, with negativity being a stronger driver of the sharing of articles concerning political out-group rather than in-group concepts^49^. This suggests that articles critical of political opponents receive disproportionate attention, lending credence to the argument that increased engagement with out-group content is driven by animosity^46^.

Our results indicate that social media platforms shape users’ news consumption beyond any control they might exert through content moderation^57^ or editorial decisions (such as the curation of Twitter Moments)^58,59^. A larger proportion of news articles shared on social media are negative compared to those published on traditional online news sites, meaning that those who access news directly through social media or via links embedded within social media posts could be more likely to encounter negative content. This trend may be particularly pronounced for specific news articles, including those concerning political out-group subjects. While user sharing on social media platforms might appear entirely voluntary, it could be influenced by platform dynamics. These dynamics are evident in the higher retweets for negative articles (see, “Variation”), a trend potentially influenced by algorithmic personalisation enhancing the prominence of certain tweets^60^.

Further, the disproportionate number of posts sharing negative news articles to social media could influence the content produced by online news outlets. Journalists working for such outlets commonly use social media^61^, often at the behest of their employers^62^. It has previously been contended that journalists’ content is affected by social media in various ways, including direct interactions with other users^63^, the monitoring of public perceptions^64^, and the discovery of newsworthy content^65^. We propose that content from journalists might also be shaped through them identifying the heightened spread of their negative articles on social media. Even journalists who are not active on social media platforms may notice spikes in the readership of negative articles driven by readers accessing articles through links embedded in social media posts. The news media is focusing increasingly on adverse events^66,67^, and our findings suggest that social media stands to exacerbate this trend: news sharing on social media could incentivise journalists to create more negative content, potentially leading to increased negative news exposure even for those who stay informed only using online news sites.

The findings presented in this study are limited by data scope. Our social media data was confined to two popular news-sharing platforms (Facebook and Twitter), thereby excluding emerging mediums for news dissemination (such as TikTok)^68^. Additionally, news article data was sourced exclusively from the NOW corpus. This corpus offers expedient access to numerous online articles but does not encompass the entirety of digital news content. Further, this news article data concerned four US- or UK-based sources, potentially missing variations in negativity bias in different countries and among readers of alternate publications^69^. However, to our knowledge, this research remains broader in scope than the existing literature on negative news sharing to social media, which has often concerned just a single social media site^29^, country^5^, or news topic^28,30^.

This research is also susceptible to methodological limitations. Social media sharing data for Facebook and Twitter were captured using different approaches (see, “Social Media Data”), potentially constraining direct comparisons between the results for each platform (see, “Variation”). Facebook post counts were obtained using an API-based approach, while tweets were captured using a script to query Twitter through its “Advanced search” feature. As a large distributed system, Twitter faces challenges with storing and promptly disseminating data requested by users. This may have resulted in the incomplete retrieval of relevant tweets, although our method yielded consistent results across multiple trials. Additionally, our analyses employed a Vader dictionary-based approach to categorise news sentiment. While such methods are widely employed^70,71^, they can suffer from imprecision^24^. Moreover, we acknowledge that our findings could be affected by factors beyond (human) user actions. For instance, bot activity could have artificially increased the share counts of certain articles^72^.

There are several potential avenues for further research, many of which would be facilitated by gathering more granular data about the individual social media users sharing news articles. For instance, investigation into out-group animosity might be advanced by considering individuals’ political viewpoints. While these viewpoints are typically aligned with their chosen news source^73,74^, they can still exhibit variation^75^. Additionally, capturing background information on news sharers could permit the exploration of between-user differences in sharing^69^ and the subsequent re-posting of shared articles^71^.

Furthermore, future work could benefit from continued collaboration with social media platforms. Previous studies have used experimental designs to explore the effects of modifying Facebook user feeds to employ a reverse-chronological algorithm^76^ and exclude re-posts of Facebook posts^56^. Given that prior research typically emphasises the negative consequences of negative news exposure (“Introduction”), it is proposed that future collaborations examine interventions to reduce the prominence of negative news articles. If successful, social media platforms could even consider this a viable long-term strategy. While social media platforms are a vital news source for many, news comprises only a fraction of the diverse content disseminated on these platforms^77^. Thus, interventions focused solely on news content might not markedly diminish users’ overall site satisfaction, unlike wholesale algorithmic changes that have prompted users to shift to competing social media sites^76^.

## Conclusion

The negativity of a news article influences its introduction to social media through user posts. While the impact of negativity might be amplified by accounting for the re-posting of user posts or moderated by an article’s political group focus, our comprehensive analysis of large-scale data samples reveals a substantial overall effect. This indicates that individuals are more likely to encounter negative news articles when accessing content on social media or through links embedded in posts. Additionally, the heightened sharing of negative articles to social media may incentivise journalists to write more negatively, potentially resulting in increased negative news exposure even for individuals who rely solely on online news sites.

## Methods

### Data

We use digital log data from social media and online news sites, which offer numerous advantages. They permit investigation into natural behaviour instead of engagement with fabricated Twitter timelines^7^ or researcher-created news headlines^69^. Additionally, they do not depend on survey responses, which can contradict news consumer behaviour^3^. Our selected data sources also provide a large sample, an important consideration when establishing overarching patterns among news consumers, given individuals’ variation in negativity bias^69^.

#### News article data

Online news articles were gathered from the News On the Web (NOW) corpus, which contains 18.2 billion words of data (as of November 2023)^78^ from newspaper and magazine articles across 20 countries^79^. We used information from online articles published across three years (2019–21) on four mainstream US and UK news sites: the Guardian, Daily Mail, New York Times, and New York Post. Our sample excluded any article containing fewer than 100 words in total or 10 words featuring in a dictionary of positive and negative terms^80^.

A binary negativity value was assigned to each article through a multi-stage process. The dictionary-based Vader sentiment tool^80^ was applied to all words in the article, producing a non-zero sentiment value (above zero for positive sentiment, below zero for negative) for words available in the Vader dictionary and a value of zero for words not available in the dictionary. Next, the sum of all non-zero sentiment scores was divided by the number of words with non-zero sentiment. If the resulting value was below zero, the article was classified as negative; otherwise, the article was classified as positive. There were no instances in which the resultant value was precisely zero. We employed a binary indicator of negativity instead of a continuous sentiment measure to promote the legibility of results, allowing us to report metrics such as the percentage increase in shares for negative articles. Dictionary-centred methods might be considered coarse means of estimating the sentiment of media sources^24^. However, the approach avoids employing a topic-based definition of negativity that may obstruct the application of topic controls (see, “Robustness”) by conflating the negativity of an article with its subject matter^29^. Further, our method draws on a valence dictionary that is widely applied (including to online news articles)^70^, is readily reproducible, and, crucially, is compatible with the NOW corpus. This corpus obstructs the use of more involved sentiment measures that require complete sentences or multi-word phrases, as 10 out of every 200 words in each NOW Corpus article are replaced with an “@” symbol.

Article text and metadata permitted the creation of numerous control variables. These encompassed various core article characteristics, such as dummy variables derived from year, month, and day-of-the-week metadata; as well as article-level calculations of the number of letters in each word, the average number of words in each sentence, and the number of words in total. All variables produced through article-level calculations were log + 1 transformed before use in inferential models. Dummy variables for the article topic were also generated through topic modelling, a data-driven approach that categorises extensive text data into cohesive topics (SI7).

Lastly, we searched for out-group and in-group political references in news articles. To achieve this, we first identified the number of instances in which each news article employed a liberal or conservative reference. Liberal references comprised liberal identity terms and the full names of serving and well-known Labour politicians (for UK papers) or Democrat politicians (for US papers). Conservative references included conservative identity terms and the full names of serving and well-known Conservatives (UK) or Republicans (US). Following Rathje, van Bavel and van der Linden^46^, prominent politicians were identified from lists of the top 100 most famous Democrat, Republican, Labour and Conservative politicians on YouGov^81^. Lists of conservative and liberal identity terms were taken directly from previous work^46,47^. In accordance with AllSides’ Media Bias Chart, the Daily Mail and New York Post were categorised as right-leaning papers, and the Guardian and New York Times were classified as left-leaning^50^. News articles were said to reference the out-group when over half of political references were out-group focused, and the in-group when over half of political references were in-group focused. These classifications therefore reflect the perceived political standpoint of news sites, which correlate with those of their readers^73,74^.

#### Social media data

The number of Facebook shares for each news article was established using Sharescore^82,83^, a social media monitoring tool providing access to information sourced from the Facebook Graph API. Through Sharescore, we searched for posts containing the URL of any news articles in our news article data. This process revealed the total number of posts concerning each news article from any Facebook account, offering a more holistic perspective than confining the data to, for example, official news site accounts^7,51^.

We implemented a custom-written solution for Twitter data, leveraging Twitter's “Advanced search” feature. This allowed us to establish the count of tweets that included the URL of any news article within our sample. In addition to retrieving the total count of tweets from any account sharing each news article, this approach provided key engagement metrics (e.g., retweet count) and the full tweet text. Possessing the full tweet allowed us to conduct sentiment analysis akin to our analysis of news articles (Table 1). The count of posts concerning each news article was log + 1 transformed before inferential analyses.

**Table 1:** Data sources summary.

News source	Data source	Number of documents	Mean words per document	Mean Vader words per document	Mean document sentiment	Proportion of documents negative	Mean Facebook posts about article	Mean tweets about article	Mean tweets and retweets about article	Mean tweet retweets	Mean tweet replies	Mean tweet likes
Daily mail	News articles	15,881	871.211	59.08	0.047	0.415	5650.936	16.246	67.903			
	Facebook posts	89,742,513										
	Tweets	258,157	16.672	1.492						3.179	1.06	6.287
	Tweets Vader	180,444	19.191	2.135	-0.114	0.646				3.315	1.208	6.813
Guardian	News articles	12,026	924.36	62.744	0.07	0.29	2671.536	50.242	210.641			
	Facebook posts	32,127,893										
	Tweets	604,212	19.244	1.6						3.193	0.605	8.431
	Tweets Vader	422,126	22.954	2.29	-0.016	0.519				3.772	0.684	9.83
New York Post	News articles	33,669	482.858	31.084	0.038	0.399	2525.039	14.723	85.498			
	Facebook posts	85,015,524										
	Tweets	495,777	14.589	1.253						4.807	3.109	13.318
	Tweets Vader	304,700	18.842	2.038	-0.095	0.615				5.489	3.475	15.298
New York Times	News articles	33,706	1216.875	76.29	0.056	0.33	10,867.595	137.514	903.802			
	Facebook posts	366,303,163										
	Tweets	4,635,405	17.648	1.4						5.572	1.353	15.542
	Tweets Vader	2,985,738	22.026	2.173	-0.016	0.518				7.206	1.729	19.866

Summary statistics for all data employed in the analysis prior to transformation. Information on the “Mean document sentiment” and “Proportion of documents negative” for tweets is provided only for subsets containing one or more words in the Vader dictionary: the “Tweets Vader” data sources. This approach was adopted to prevent these statistics from conveying an inaccurate impression of neutrality, which may have arisen because certain tweets contain minimal text. Summary statistics for data used to produce aggregate findings (reported in “Article Negativity and Sharing to Social Media”, and “Article Negativity, Out-Group Content, and Sharing to Social Media”) are provided in SI10.

#### Data summary

Key summary statistics are provided (Table 1). These show that the number of Facebook shares for articles vastly exceeds the number of Twitter shares. This disparity belies a strong correlation between the count of posts and tweets for each article (SI8). Negative news articles are in the minority for all news sites (29% to 42%)^84,85^. We also note that news articles are consistently less negative than the tweets that concern them, with all tweet samples being predominantly negative^85,86^.

### Analyses

H1 was investigated using Doubly Robust Estimation (DRE), which integrates elements from both Multiple Regression (MR) and Propensity Score (PS) methods (SI9)^87^. MR and PS approaches are each effective in accounting for confounders on their own (and were therefore employed in alternate models, see, “Robustness”). However, incorporating elements from both approaches within DRE yields an unbiased treatment effect estimate under the misspecification of either the MR or PS component. A point estimate and 95% confidence intervals were established by applying DRE to 1000 bootstrap samples. The use of bootstrapping renders the DRE model robust even in the absence of normality assumptions. Nonetheless, we verify normality through a histogram of log(+ 1) transformed social media shares and a Quantile–Quantile (QQ) plot of model residuals obtained from an MR model (SI11).

H2 was explored through MR models specified with an interaction term between variables showing whether a news article was negative and concerned with political out-group concepts (see, “Article Negativity, Out-Group Content, and Sharing to Social Media”). In this case, our model p-values assume normality, which we confirm through a histogram of log(+ 1) social media shares and a QQ plot of model residuals (SI11).

## Supplementary Information

Supplementary Information.

## Data Availability

The data compiled and analysed during this research, along with the scripts used for data analysis, have been made available at https://github.com/JoeMarkWatson/negative_news_sharing.
