# Supplementary material for: Negative online news articles are shared more to social media
Source: Sci Rep. 2024 Sep 16;14:21592. doi: 10.1038/s41598-024-71263-z (PMC11405697; doi:10.1038/s41598-024-71263-z)
Supplement: Supplementary file 1 — Supplementary Information. [file 41598_2024_71263_MOESM1_ESM.docx]

**Nature Scientific Reports: Supplementary information file**

Negative online news articles are shared more to social media

Joe Watson^a^*, Sander van der Linden^b^, Michael Watson^c^, David Stillwell^a,d^

^a^Psychometrics Centre, Judge Business School, University of Cambridge, UK
^b^Department of Psychology, University of Cambridge, UK
^c^Department of Informatics, King’s College London, UK
^d^Organisational Behaviour Group, Judge Business School, University of Cambridge, UK

*To whom correspondence may be addressed. Email: [j.watson@jbs.cam.ac.uk](mailto:j.watson@jbs.cam.ac.uk)

**Keywords**: Negativity Bias, Online News, Social Media

# Supplementary information

## SI 1: Effect Estimates for Negativity

**Table SI.1.1.** Negativity effect estimates for models presented in Fig. 1 and Fig. SI.3.1

| Method | Outcome Variable | News Site | Sample | Point Estimate | Lower Bound | Upper Bound |
| --- | --- | --- | --- | --- | --- | --- |
| DRE | Tweets | DM | 15,881 | 0.232 | 0.197 | 0.268 |
|  |  | G | 12,026 | 0.309 | 0.262 | 0.354 |
|  |  | NYP | 33,669 | 0.269 | 0.250 | 0.289 |
|  |  | NYT | 33,706 | 0.254 | 0.223 | 0.286 |
|  | Facebook posts | DM | 15,881 | 0.566 | 0.481 | 0.659 |
|  |  | G | 12,026 | 0.265 | 0.156 | 0.370 |
|  |  | NYP | 33,669 | 0.916 | 0.860 | 0.970 |
|  |  | NYT | 33,706 | 0.567 | 0.517 | 0.618 |
| DRE topic control | Tweets | DM | 15,881 | 0.217 | 0.180 | 0.252 |
|  |  | G | 12,026 | 0.226 | 0.182 | 0.270 |
|  |  | NYP | 33,669 | 0.151 | 0.131 | 0.172 |
|  |  | NYT | 33,706 | 0.230 | 0.198 | 0.262 |
|  | Facebook posts | DM | 15,881 | 0.514 | 0.415 | 0.613 |
|  |  | G | 12,026 | 0.154 | 0.039 | 0.261 |
|  |  | NYP | 33,669 | 0.421 | 0.363 | 0.480 |
|  |  | NYT | 33,706 | 0.470 | 0.414 | 0.523 |
| DRE tweets and retweets | Tweets plus retweets | DM | 15,881 | 0.331 | 0.285 | 0.378 |
|  |  | G | 12,026 | 0.463 | 0.403 | 0.528 |
|  |  | NYP | 33,669 | 0.391 | 0.365 | 0.419 |
|  |  | NYT | 33,706 | 0.508 | 0.465 | 0.554 |
| MR | Tweets | DM | 15,881 | 0.232 | 0.198 | 0.265 |
|  |  | G | 12,026 | 0.282 | 0.240 | 0.325 |
|  |  | NYP | 33,669 | 0.250 | 0.231 | 0.268 |
|  |  | NYT | 33,706 | 0.232 | 0.205 | 0.260 |
|  | Facebook posts | DM | 15,881 | 0.557 | 0.474 | 0.641 |
|  |  | G | 12,026 | 0.240 | 0.148 | 0.332 |
|  |  | NYP | 33,669 | 0.846 | 0.793 | 0.898 |
|  |  | NYT | 33,706 | 0.536 | 0.488 | 0.584 |
| PS | Tweets | DM | 15,881 | 0.225 | 0.189 | 0.261 |
|  |  | G | 12,026 | 0.248 | 0.198 | 0.296 |
|  |  | NYP | 33,669 | 0.173 | 0.152 | 0.196 |
|  |  | NYT | 33,706 | 0.197 | 0.160 | 0.233 |
|  | Facebook posts | DM | 15,881 | 0.557 | 0.470 | 0.650 |
|  |  | G | 12,026 | 0.150 | 0.041 | 0.250 |
|  |  | NYP | 33,669 | 0.699 | 0.640 | 0.757 |
|  |  | NYT | 33,706 | 0.460 | 0.406 | 0.513 |
| DRE alternate treatment | Tweets | DM | 15,881 | 0.233 | 0.198 | 0.268 |
|  |  | G | 12,026 | 0.260 | 0.218 | 0.297 |
|  |  | NYP | 33,669 | 0.258 | 0.240 | 0.277 |
|  |  | NYT | 33,706 | 0.253 | 0.225 | 0.280 |
|  | Facebook posts | DM | 15,881 | 0.524 | 0.436 | 0.620 |
|  |  | G | 12,026 | 0.226 | 0.136 | 0.316 |
|  |  | NYP | 33,669 | 0.857 | 0.803 | 0.908 |
|  |  | NYT | 33,706 | 0.520 | 0.474 | 0.565 |

Results from each model were created using DRE, MR or PS methods to calculate the impact of negativity on a log(+1) transformed outcome variable. For all methods except the DRE alternate treatment approach, a negative article is one with a sentiment score below zero (*News Article Data*). For the DRE alternate treatment approach only, a negative article is one with a sentiment score below the mean sentiment score of all articles. All methods controlled for article characteristics (*Methods*) only, except the DRE topic control method that also controlled for article topic (*News Article Data*). Lower and upper bound values represent the 95% confidence intervals. Findings for any sample from our core method (DRE) only deviated from those in a robustness check method (MR, PS, DRE alternate treatment or DRE topic control) in 4 of 32 possible instances. Conversely, the estimated effect of negativity on tweets plus retweets exceeded that obtained when predicting tweets alone across all (4 of 4) news sites.

## SI 2: Effect Estimates for Aggregated Models

**Table SI.2.1.** Negativity effect estimates for aggregated primary research question models

| Outcome Variable | News Sites | Sample | Point Estimate | Lower Bound | Upper Bound |
| --- | --- | --- | --- | --- | --- |
| Tweets plus Facebook posts | All | 95,282 | 0.646 | 0.618 | 0.678 |
| Facebook posts |  | 95,282 | 0.682 | 0.649 | 0.716 |
| Tweets |  | 95,282 | 0.295 | 0.278 | 0.312 |
| Tweets plus retweets |  | 95,282 | 0.478 | 0.456 | 0.501 |
| Facebook posts | Guardian, New York Times | 45,732 | 0.531 | 0.489 | 0.576 |
|  | Daily Mail, New York Post | 49,550 | 0.799 | 0.754 | 0.843 |
| Tweets | Guardian, New York Times | 45,732 | 0.299 | 0.273 | 0.325 |
|  | Daily Mail, New York Post | 49,550 | 0.271 | 0.253 | 0.288 |

Results from each model were generated using DRE to gauge the effect of negativity on a log(+1) transformed outcome variable. All models controlled for article characteristics (*Methods*) and dummy variables representing news sites. Lower and upper bound values represent the 95% confidence intervals.

**Table SI.2.2.** Coefficient estimates for aggregated secondary research question models

| Outcome Variable | News Site | Sample | Negativity effect | Out-group effect | Interaction effect |
| --- | --- | --- | --- | --- | --- |
| Tweets and Facebook posts | All | 24,828 | 0.341* | 0.139* | 0.195* |

Values were estimated using MR specified with an interaction effect. This MR model also controlled for article characteristics (*Methods*) and dummy variables representing news sites. An asterisk, “*”, is used to signify significant coefficient values at p = 0.05. The lower and upper bound values representing the 95% confidence intervals have been omitted from this table to promote legibility. They are available at: <https://github.com/JoeMarkWatson/negative_news_sharing/blob/main/data/all_results.csv>.

## SI 3: Plot of Negativity Impact for Additional Models


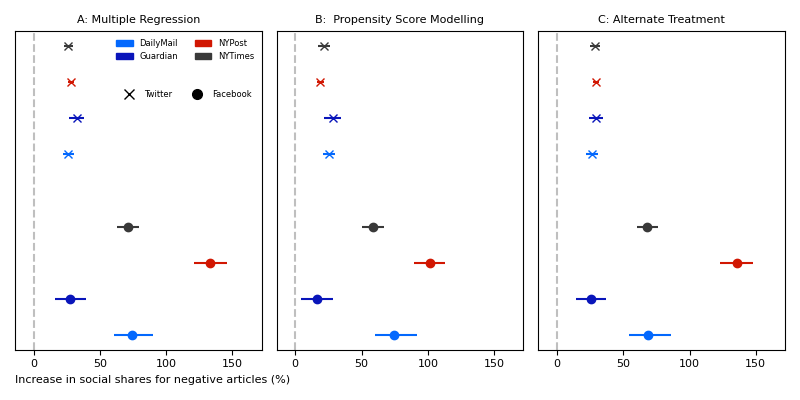


**Fig. SI.3.1.** Point estimates and error bars (95% confidence intervals) for the effect of article negativity on log(+1) article shares, converted into percentage increases to enhance readability (*SI 9*). For the MR (*A*) and PS (*B*) approaches, a negative article is one with a sentiment score below zero (*News Article Data*). For the Alternate Treatment approach (*C*) that uses DRE, a negative article is one with a sentiment score below the mean sentiment score of all articles. Treatment effect values are provided in *SI 1*. The effect of article negativity on shares is positive and significant across every dataset when applying MR (*A*), PS (*B*), and DRE with an alternate treatment variable (*C*, *News Article Data*).

## SI 4: Effect Estimates for Models Predicting Retweets, Comments and Likes

**Table SI.4.1.** Negativity effect estimates for tweet reactions

| Sample | Outcome Variable | Point Estimate | Lower Bound | Upper Bound |
| --- | --- | --- | --- | --- |
| 5,992,982 | Retweets | 0.022 | 0.021 | 0.024 |
|  | Comments | 0.009 | 0.008 | 0.01 |
|  | Likes | -0.025 | -0.027 | -0.023 |

Results from each model were generated using MR to gauge the effect of the negativity of the news article linked in a tweet on a log(+1) transformed outcome variable. All models controlled for article characteristics (*Methods*), tweet character length and dummy variables representing the news site of the URL linked in the original tweet. Lower and upper bound values represent the 95% confidence intervals.

## SI 5: Effect Estimates for Negativity When Dividing by Topic

**Table SI.5.2.** Negativity effect estimates for models presented in Fig. 2

| Outcome Variable | News Site | Topic | Sample | Point Estimate | Lower Bound | Upper Bound |
| --- | --- | --- | --- | --- | --- | --- |
| Tweets | DM | Politics | 1,385 | 0.261 | 0.148 | 0.374 |
|  |  | Family and home | 7,210 | 0.245 | 0.195 | 0.295 |
|  |  | Sports | 1,378 | -0.162 | -0.286 | -0.039 |
|  |  | Local news | 3,887 | 0.263 | 0.193 | 0.334 |
|  |  | Global news | 2,021 | 0.182 | 0.083 | 0.281 |
|  | G | Politics | 1,213 | 0.125 | 0.000 | 0.250 |
|  |  | Family and home | 3,970 | 0.185 | 0.110 | 0.259 |
|  |  | Sports | 2,068 | 0.274 | 0.148 | 0.401 |
|  |  | Local news | 1,215 | 0.155 | 0.037 | 0.273 |
|  |  | Global news | 3,560 | 0.267 | 0.194 | 0.339 |
|  | NYP | Politics | 5,074 | 0.119 | 0.069 | 0.169 |
|  |  | Family and home | 4,531 | 0.266 | 0.210 | 0.321 |
|  |  | Sports | 10,191 | 0.045 | 0.015 | 0.076 |
|  |  | Local news | 8,487 | 0.141 | 0.102 | 0.179 |
|  |  | Global news | 5,386 | 0.187 | 0.139 | 0.235 |
|  | NYT | Politics | 8,428 | 0.220 | 0.167 | 0.273 |
|  |  | Family and home | 6,644 | 0.253 | 0.187 | 0.320 |
|  |  | Sports | 2,377 | 0.234 | 0.104 | 0.363 |
|  |  | Local news | 7,288 | 0.233 | 0.180 | 0.286 |
|  |  | Global news | 8,969 | 0.143 | 0.087 | 0.200 |
| Facebook posts | DM | Politics | 1,385 | 0.252 | 0.000 | 0.505 |
|  |  | Family and home | 7,210 | 0.751 | 0.623 | 0.879 |
|  |  | Sports | 1,378 | -0.580 | -1.011 | -0.150 |
|  |  | Local news | 3,887 | 0.536 | 0.364 | 0.708 |
|  |  | Global news | 2,021 | 0.355 | 0.141 | 0.568 |
|  | G | Politics | 1,213 | 0.147 | -0.111 | 0.405 |
|  |  | Family and home | 3,970 | -0.063 | -0.229 | 0.103 |
|  |  | Sports | 2,068 | 0.148 | -0.180 | 0.476 |
|  |  | Local news | 1,215 | -0.007 | -0.277 | 0.263 |
|  |  | Global news | 3,560 | 0.350 | 0.195 | 0.504 |
|  | NYP | Politics | 5,074 | 0.304 | 0.179 | 0.429 |
|  |  | Family and home | 4,531 | 0.832 | 0.687 | 0.978 |
|  |  | Sports | 10,191 | 0.017 | -0.097 | 0.132 |
|  |  | Local news | 8,487 | 0.594 | 0.490 | 0.699 |
|  |  | Global news | 5,386 | 0.535 | 0.411 | 0.660 |
|  | NYT | Politics | 8,428 | 0.259 | 0.167 | 0.350 |
|  |  | Family and home | 6,644 | 0.355 | 0.242 | 0.468 |
|  |  | Sports | 2,377 | 0.320 | 0.018 | 0.622 |
|  |  | Local news | 7,288 | 0.762 | 0.665 | 0.858 |
|  |  | Global news | 8,969 | 0.490 | 0.398 | 0.583 |

All negativity effect estimates were calculated using MR models controlling for article characteristics (*Methods*). Lower and upper bound values represent the 95% confidence intervals.

## SI 6: Effect Estimates for Negativity, Out-group Referencing and Their Interaction

**Table SI.6.1** Coefficient estimates for models presented in Fig. 3

| Outcome Variable | News Site | Sample | Negativity effect | Out-group effect | Interaction effect |
| --- | --- | --- | --- | --- | --- |
| Tweets | DM | 2,701 | 0.118* | -0.099 | 0.221* |
|  | G | 3,185 | 0.168* | 0.098 | -0.020 |
|  | NYP | 6,913 | 0.127* | -0.003 | 0.130* |
|  | NYT | 12,029 | 0.184* | 0.164* | 0.039 |
| Facebook posts | DM | 2,701 | 0.242 | 0.099 | 0.384* |
|  | G | 3,185 | -0.04 | -0.021 | 0.265 |
|  | NYP | 6,913 | 0.335* | -0.103 | 0.382* |
|  | NYT | 12,029 | 0.376 | 0.307* | 0.163* |

Coefficient effect estimates were calculated using MR models that controlled for article characteristics (*Methods*) and included an interaction effect between negativity and political out-group referencing. An asterisk, “*”, is used to signify coefficient values that are significant at p = 0.05. Lower and upper bound values representing the 95% confidence intervals have been omitted from this table to promote readability. These are provided at: <https://github.com/JoeMarkWatson/negative_news_sharing/blob/main/data/all_results.csv>.

## SI 7: Most Influential Terms Associated with Topic Classifications

We considered several competing topic models, which separated the data into differing numbers of topics. These models were all constructed using the 2000 most frequent terms in all newspaper articles, ensuring that each term did not appear in more than 85% of documents and was not part of a predefined list of stop words. The list of stop words comprised common terms like "also" and "the" – typically considered uninformative in capturing text content – and all terms in the Vader sentiment dictionary. Including Vader terms could otherwise have introduced multicollinearity into our regression models, as the article topic might then influence the negativity variable. Ultimately, we selected a 5-topic model due to its superior log likelihood score compared to models with fewer topics and its production of coherent output. These topics were labelled as 'global,' 'local,' 'family and home,' 'politics,' and 'sports' news, aligning with the most influential terms associated with each topic classification (Table SI.7.1).

**Table SI.7.1** Ten most influential terms associated with each topic

|  | Politics | Family and home | Sports | Local news | Global news |
| --- | --- | --- | --- | --- | --- |
| Word 0 | president | time | game | police | people |
| Word 1 | trump | family | season | mr | health |
| Word 2 | mr | people | team | people | year |
| Word 3 | state | years | games | york | coronavirus |
| Word 4 | house | life | time | city | government |
| Word 5 | biden | children | players | times | percent |
| Word 6 | election | home | year | man | vaccine |
| Word 7 | people | women | league | time | york |
| Word 8 | states | day | player | officers | company |
| Word 9 | government | way | way | years | city |
| Word 10 | country | school | week | image | virus |

## SI 8: Correlation Between Facebook and Twitter Shares


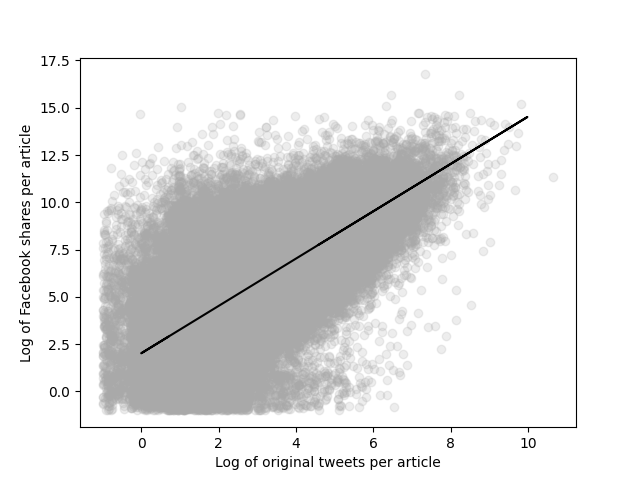


**Fig. SI.8.1.** Scatter plot with correlation line showing a positive correlation between the log(+1) count of tweets and log(+1) count of Facebook posts concerning all news articles (from every online news site sample). This correlation is strong and significant, with a coefficient of 0.688 (p = 0.00). The points on the scatter plot are randomly jittered to reduce overlap, providing a clearer representation of the data distribution.

## SI 9: Further Details on Analyses

Our implementation of DRE employs two distinct linear models that regress our outcome variable, $Y$, on covariates, $X$. One is fit using exclusively treated samples (meaning negative articles), while another is fit using untreated samples (meaning positive articles). These models generate predictions of the outcome $\mu_{1}\left( X_{i} \right)$ and $\mu_{0}\left( X_{i} \right)$ respectively. The approach also accounts for each sample’s propensity for treatment. We estimate the relationship between covariates, $X$, and treatment indicator, $T$, using logistic regression and then predict for each sample to find propensity score $\hat{P}\left( X_{i} \right)$. The average treatment effect, $A\hat{T}E$, is then calculated using DRE in the following manner (Formula 1):

(1)

$$A\hat{T}E=\frac{1}{N} \sum_{i=1}^{N} \left( \frac{T_{i}\left( Y_{i}-\mu_{1}\left( X_{i} \right) \right)}{\hat{P}\left( X_{i} \right)}+\mu_{1}\left( X_{i} \right) \right)-\frac{1}{N} \sum_{i=1}^{N} \left( \frac{\left( 1-T_{i} \right)\left( Y_{i}-\mu_{0}\left( X_{i} \right) \right)}{1-\hat{P}\left( X_{i} \right)}+\mu_{0}\left( X_{i} \right) \right)$$

A point estimate and 95% confidence intervals were constructed by performing this calculation on 1,000 bootstrap samples. For each sample, the calculation gives the effect size of treatment (a binary measure of article negativity, *News Article Data*) on the outcome variable (log+1 social media posts) for a given set of covariates. We interpret $A\hat{T}E$ as an approximate percentage increase in raw shares using the following conversion (Formula 2)^1^:

(2)

$$A\hat{T}E_{pct}\sim\left( exp(A\hat{T}E)-1 \right)*100$$

### Reference

1. Wooldridge, J. M. *Econometric Analysis of Cross Section and Panel Data*. (The MIT Press, 2010).

## SI 10: Summary Statistics for Aggregated Data

## **Table SI.10.** Aggregated Data Sources Summary

| News article subset | Number of documents | Mean words per document | Mean Vader words per document | Mean document sentiment | Proportion of documents negative | Mean tweets and Facebook posts about article | | Mean Facebook posts about article | | Mean tweets about article | | Mean tweets and retweets about article | |
| --- | --- | --- | --- | --- | --- | --- | --- | --- | --- | --- | --- | --- | --- |
|  |  |  |  |  |  | Mean | Stdev | Mean | Stdev | Mean | Stdev | Mean | Stdev |
| All | 95282 | 862.969 | 55.738 | 0.05 | 0.364 | 6078.609 | 53646.316 | 6015.712 | 53557.268 | 62.897 | 227.936 | 387.835 | 2333.525 |
| Political articles | 24828 | 1155.415 | 75.994 | 0.028 | 0.406 | 11368.391 | 69423.673 | 11258.664 | 69259.024 | 109.727 | 323.337 | 789.397 | 3687.844 |

Summary statistics for data employed in aggregated analyses prior to transformation. The ‘Political articles’ subset of news article is formed of articles that predominantly referenced a political out- or in-group (*News article data*).

## SI 11: Histogram and QQ Plots to Consider Normality


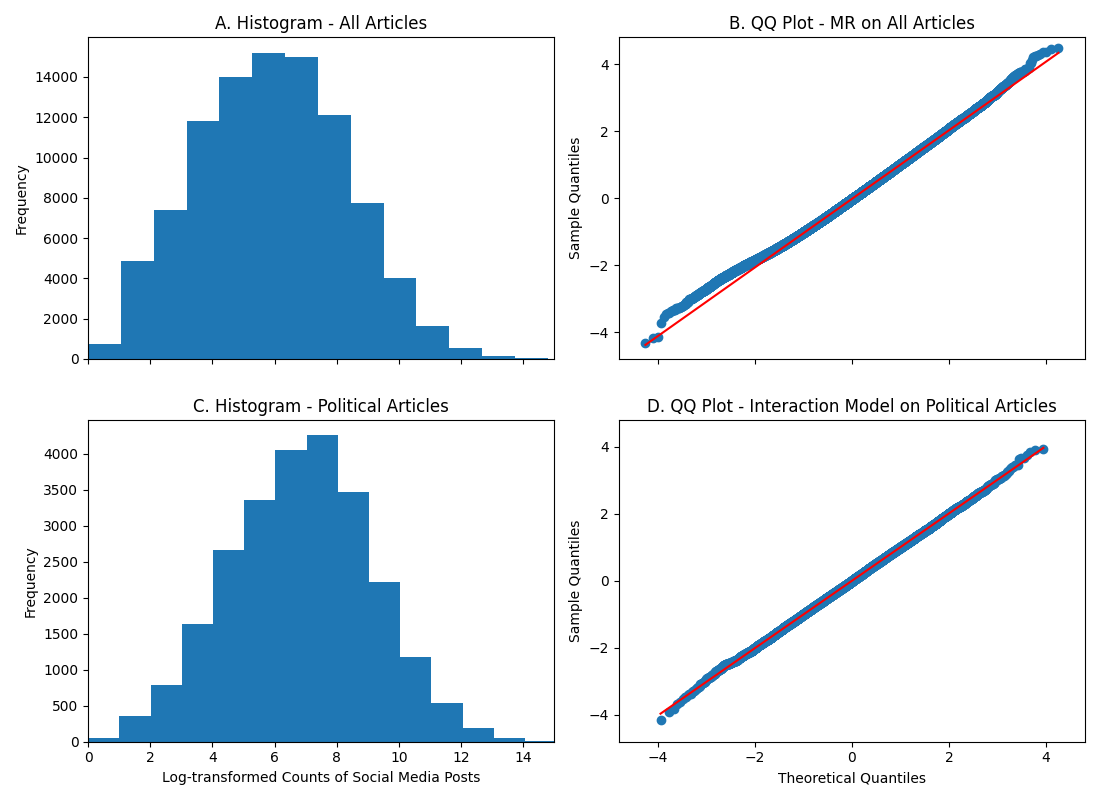


**Fig. SI.11.1.** Plots to consider the assumption of normality underlying parametric tests. *A* and *C* are histograms with 15 bins of the log-transformed counts of social media posts (tweets plus Facebook posts) received by all news articles (*A*) and all articles predominantly referencing a political out- or in-group (*C*, *News article data*). *B* and *D* are Quantile-Quantile (QQ) plots that plot model residuals against the quantiles of a theoretical normal distribution. In *B*, the model is a MR model applied to all articles (controlling for all control variables accounted for in our core DRE model, *Article Negativity and Sharing to Social Media*). In *D*, the model is the MR model with an interaction term between article negativity and out-group referencing used to investigate our secondary research questions (*Article Negativity, Out-Group Content, and Sharing to Social Media*). We interpret these plots as indicative of normality, as evidenced by the bell-shaped curves of both histograms (*A*, *C*) and alignment of the data points along a straight line in both QQ plots (*B*, *D*).
